# Supplementary material for: Novel Virus-Like Particle Vaccine Encoding the Circumsporozoite Protein of Plasmodium falciparum Is Immunogenic and Induces Functional Antibody Responses in Mice
Source: Front Immunol. 2021 Mar 17;12:641421. doi: 10.3389/fimmu.2021.641421 (PMC8010251; doi:10.3389/fimmu.2021.641421)
Supplement: Supplementary file 1 [file DataSheet_1.docx]

# Supplementary: Novel virus-like particle vaccine encoding the circumsporozoite protein of *Plasmodium falciparum* is immunogenic and induces functional antibody responses in mice

### AUTHORS AND AFFILIATIONS

Liriye Kurtovic^1,2#^, David Wetzel^3#^, Linda Reiling^1^, Damien R. Drew^1^, Catherine Palmer^1^, Betty Kouskousis^1^, Eric Hanssen^4^, Bruce D. Wines^1,2,5^, P. Mark Hogarth^1,2,5^, Manfred Suckow^3^, Volker Jenzelewski^3^, Michael Piontek^3^, Jo-Anne Chan^1,2,6#*^, James G. Beeson^1,2,6,7#*^

^1^Burnet Institute, Melbourne, Australia.

^2^Departments of Immunology and Pathology and Infectious Diseases, Central Clinical School, Monash University, Melbourne, Australia.

^3^ARTES Biotechnology GmbH, Langenfeld, Germany.

^4^The Bio21 Molecular Science and Biotechnology Institute, The University of Melbourne, Parkville, Australia.

^5^Clinical Pathology, The University of Melbourne, Parkville, Australia

^6^Department of Medicine, Royal Melbourne Hospital, The University of Melbourne, Parkville, Australia

^7^Department of Microbiology, Monash University, Clayton, Australia.

^#^Authors contributed equally (co-first and co-senior authors)

*Corresponding authors: James Beeson & Jo-Anne Chan; 85 Commercial Road, Melbourne, Australia; +61 3 9282 2111; beeson@burnet.edu.au & jo-anne.chan@burnet.edu.au

###

###


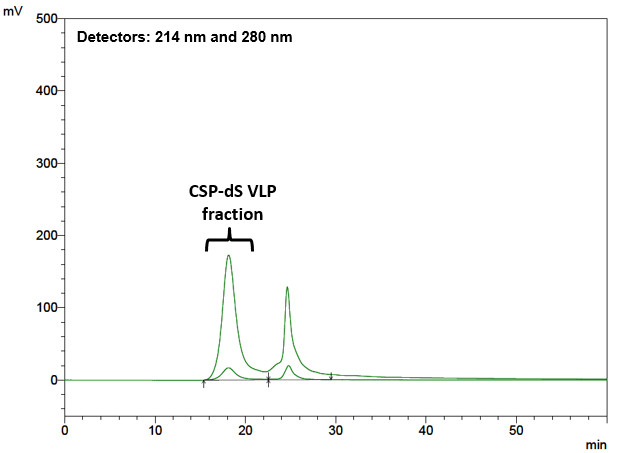

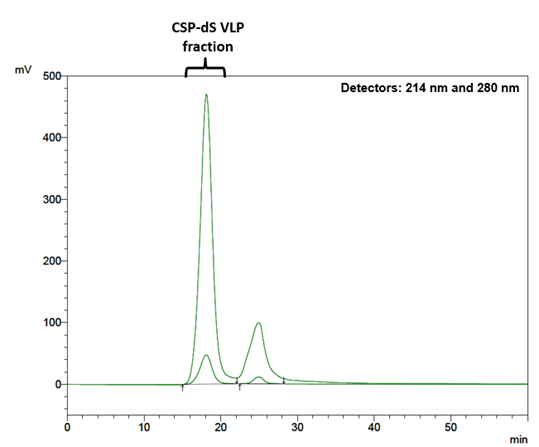


**A**

**B**

**Figure S1. Chromatogram of the HP-SEC analysis of the CSP-dS VLP preparation.** (**A**) Elution profiles were recorded at 214 nm (peptide bonds) and 280 nm (aromatic amino acids). Fractions were analyzed by SDS-PAGE (data not shown) and the marked peak was identified as product-containing fraction and analyzed by DLS. (**B**) HP-SEC chromatogram of material after storage for 4-8 °C for 6 months.


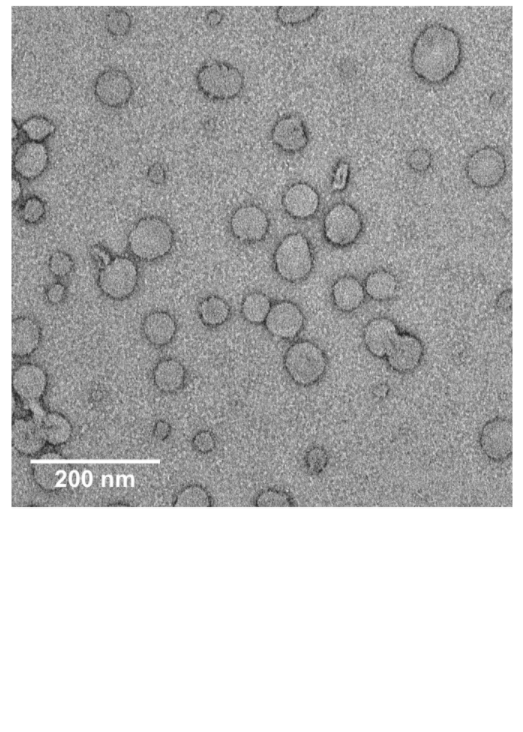

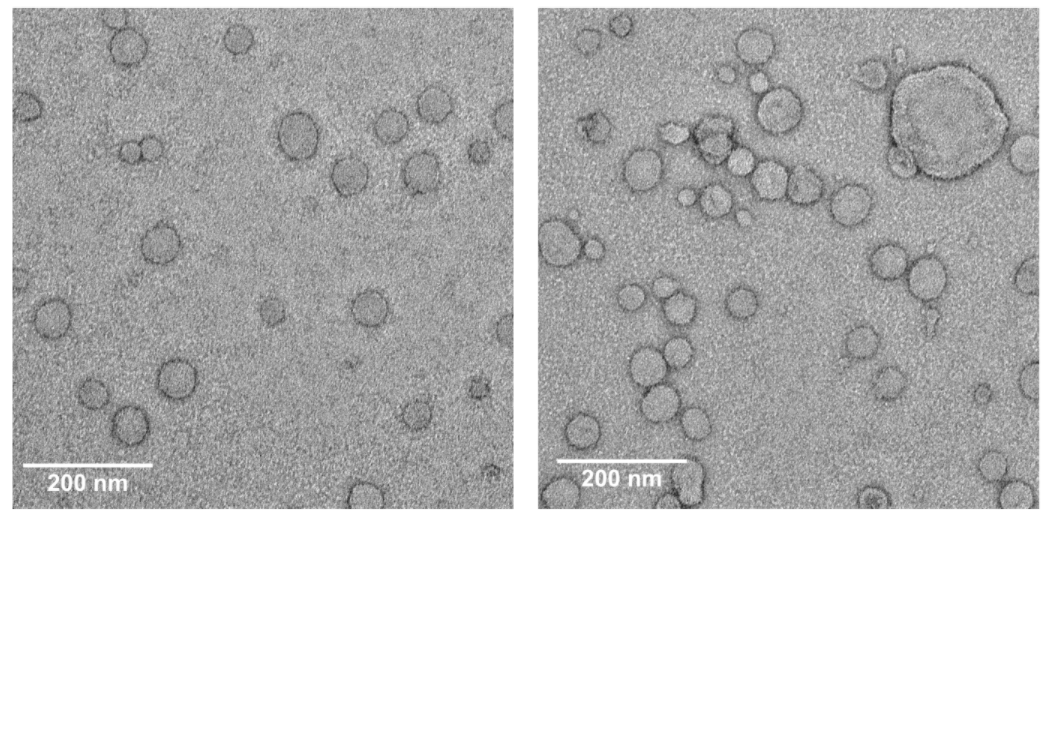


**Figure S2. Representative TEM images the structure of CSP-dS VLPs.**


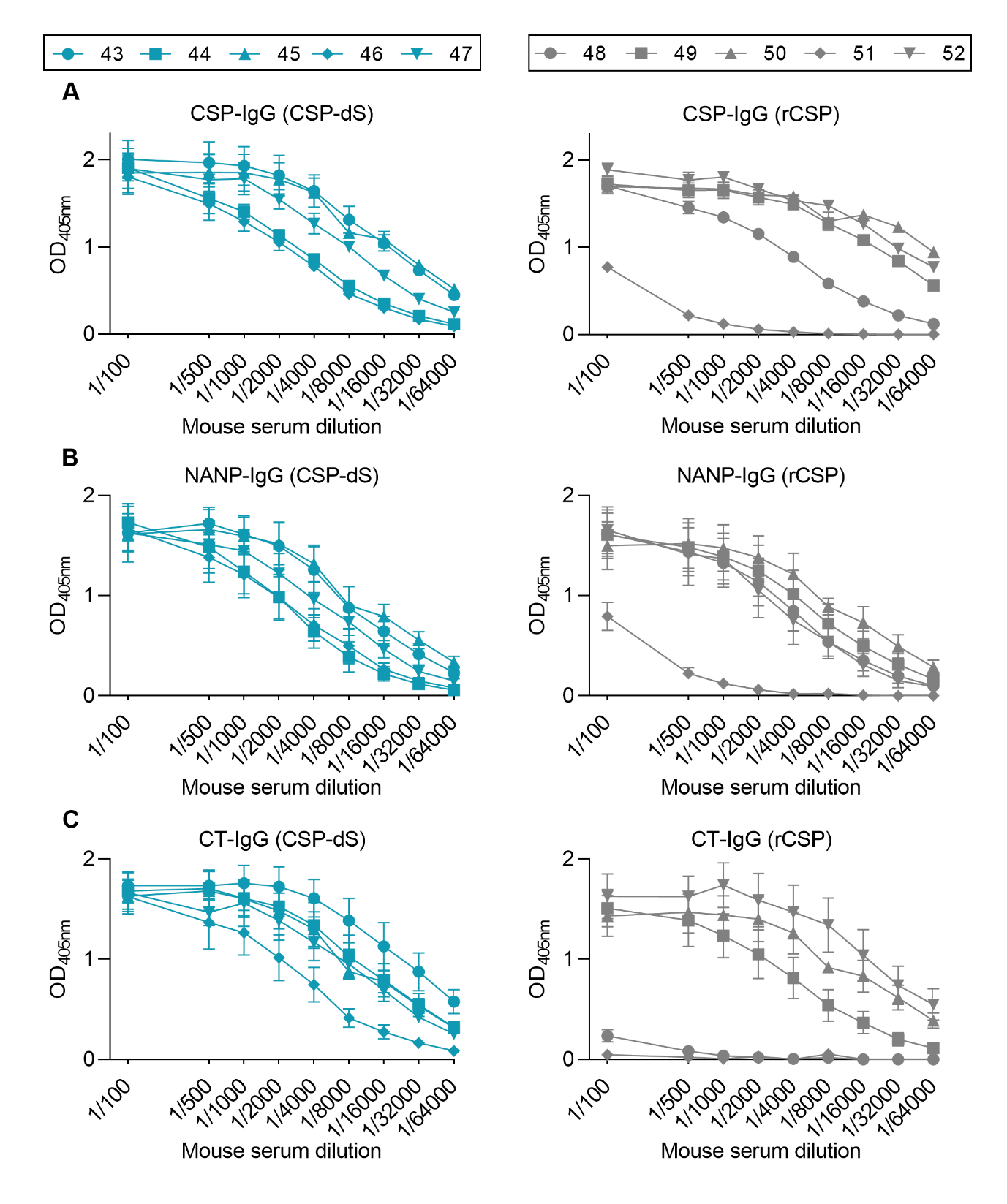


**Figure S3. Antibody data from individual mice immunized with CSP-dS and monomer vaccines.** Swiss mice were immunized with three 10 μg doses of CSP-dS (mouse #43-47) or rCSP as a control (mouse #48-52). Serum samples collected after the final immunization were tested for total IgG to (**A**) full-length CSP, and antigens representing the (**B**) central-repeat (NANP) and (**C**) C-terminal (CT) regions of CSP. The x-axis is presented on a log2 scale and the mean and range of two independent experiments are shown.


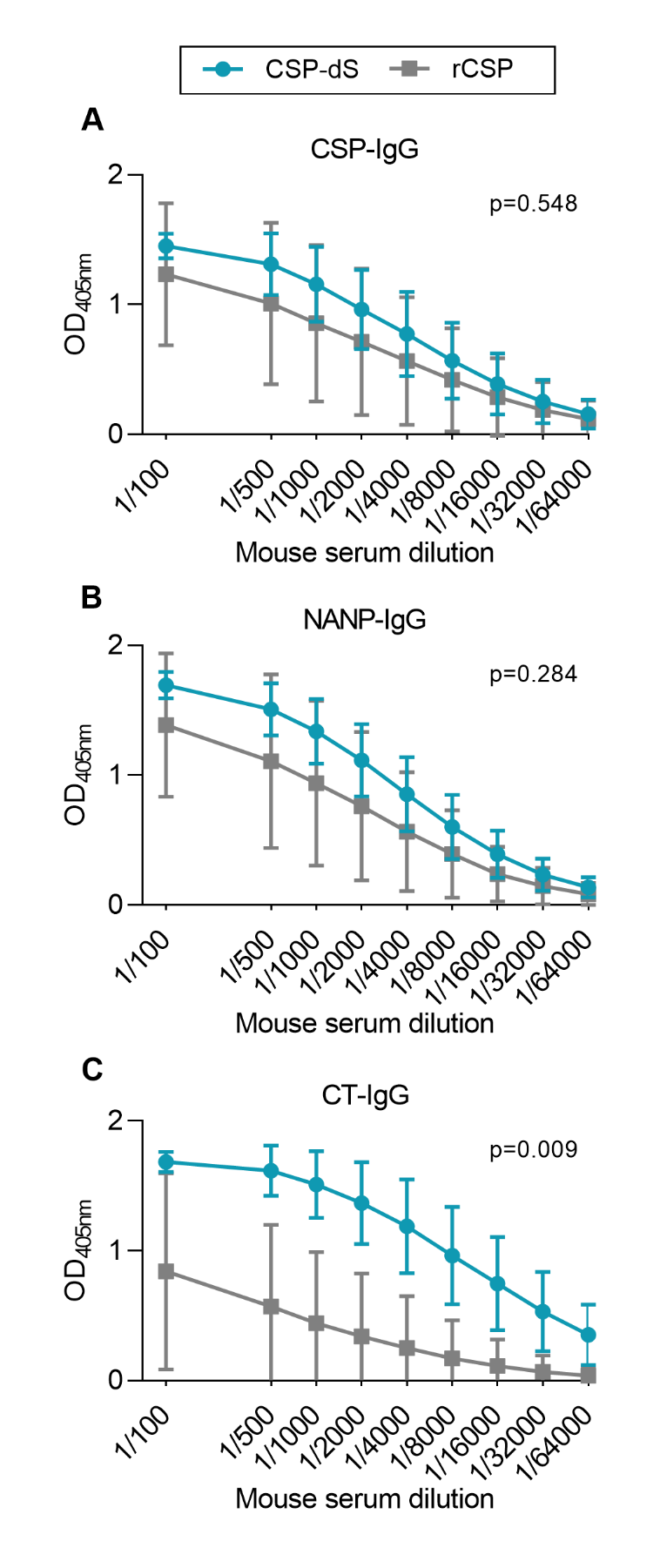


**Figure S4. Immunogenicity of CSP-dS after two doses.** Swiss mice were immunized with three 10 μg doses of CSP-dS (n=5; circles) or rCSP as a control (n=5; squares). Serum samples collected after the second immunization were tested for total IgG to (**A**) full-length CSP, and antigens representing the (**B**) central-repeat (NANP) and (**C**) C-terminal (CT) regions of CSP. The x-axis is presented on a log2 scale and the mean and standard deviation of one experiment is shown. The AUC for each mouse in the CSP-dS and rCSP vaccine groups were compared using the unpaired t-test.


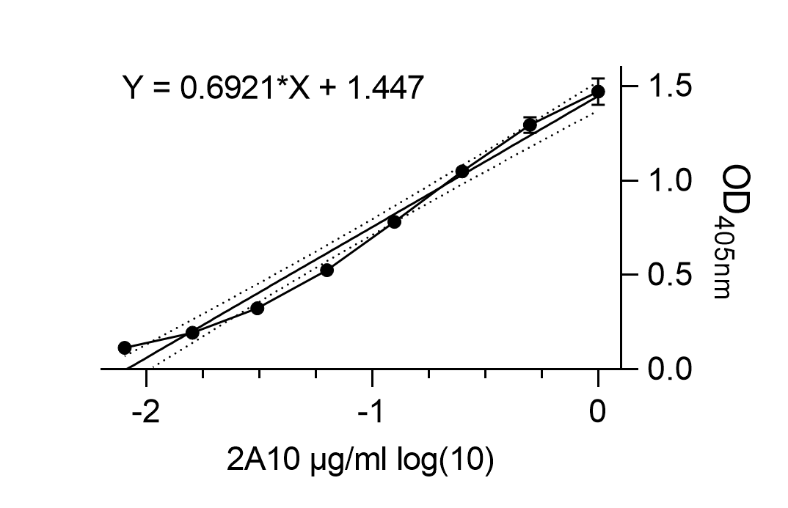


**Figure S5. Standard curve generated using the mouse 2A10 mAb.** The 2A10 mAb was tested for NANP-IgG between 1 and 0.008 μg/ml. The resulting OD values were plotted against the mAb concentration (log10 transformed) to generate an 8-point standard curve. The mean and range of duplicates are shown.
